# A generative model to simulate spatiotemporal dynamics of biomolecules in cells

**DOI:** 10.1017/S2633903X2300020X

**Published:** 2023-11-13

**Authors:** Lisa Balsollier, Frédéric Lavancier, Jean Salamero, Charles Kervrann

**Affiliations:** 1 LMJL, UMR 6629, CNRS, Nantes Université, Nantes, France; 2 SERPICO Project-Team, Centre INRIA de l’Université de Rennes, Rennes Cedex, France; 3 Institut Curie, UMR 144, CNRS, PSL Research University, Sorbonne Universités, Paris, France; 4 CREST-ENSAI, UMR CNRS 9194, Campus de Ker-Lann, Rue Blaise Pascal, Bruz Cedex, France

**Keywords:** birth-death-move process, fluorescence microscopy, intracellular dynamics and molecular motion, simulation and image synthesis, spatial statistics

## Abstract

Generators of space-time dynamics in bioimaging have become essential to build ground truth datasets for image processing algorithm evaluation such as biomolecule detectors and trackers, as well as to generate training datasets for deep learning algorithms. In this contribution, we leverage a stochastic model, called birth-death-move (BDM) point process, in order to generate joint dynamics of biomolecules in cells. This particle-based stochastic simulation method is very flexible and can be seen as a generalization of well-established standard particle-based generators. In comparison, our approach allows us: (1) to model a system of particles in motion, possibly in interaction, that can each possibly switch from a motion regime (e.g., Brownian) to another (e.g., a directed motion); (2) to take into account finely the appearance over time of new trajectories and their disappearance, these events possibly depending on the cell regions but also on the current spatial configuration of all existing particles. This flexibility enables to generate more realistic dynamics than standard particle-based simulation procedures, by for example accounting for the colocalization phenomena often observed between intracellular vesicles. We explain how to specify all characteristics of a BDM model, with many practical examples that are relevant for bioimaging applications. As an illustration, based on real fluorescence microscopy datasets, we finally calibrate our model to mimic the joint dynamics of Langerin and Rab11 proteins near the plasma membrane, including the well-known colocalization occurrence between these two types of vesicles. We show that the resulting synthetic sequences exhibit comparable features as those observed in real microscopy image sequences.

## Impact Statement

The paper presents agenerator of spatio-temporal dynamic for bio-imaging, called the birth-death-move (BDM) model. This stochastic model simulates particle dynamics, accounting for interactions and colocalization. We illustrate the high flexibility of this model by presenting results on real-word image series. Model calibration from real fluorescence microscopy data shows that it faithfully reproduces the joint dynamics of the Langerin and Rab11 proteins.

## 1. Introduction

A long-term goal in fundamental biology is to decipher the spatiotemporal dynamic coordination and organization of interacting molecules within molecular complexes at the single cell-level. This includes the characterization of intracellular dynamics, which is essential to a better understanding of fundamental mechanisms like membrane transport. To that end, dedicated image analysis methods have been developed to process challenging temporal series of 2D–3D images acquired by fluorescence microscopy.^(^^1^^)^

In this context, mathematical and biophysical models are indispensable to decode and synthesize the traffic flows of biomolecules. They constitute crucial prior models in most particle tracking procedures and they are needed to carry out simulations in order to evaluate the performance of image analysis algorithms and to facilitate the data augmentation step for the training of complex models like deep neural networks. Among them, particle-based stochastic models form the main class of tracking models^(^^2^^–^^5^^)^ and they are often at the basis of single molecule localization microscopy (SMLM) simulators.^(^^6^^–^^10^^)^ Popular softwares providing particle-based stochastic simulations include Virtual Cell,^(^^11^^)^ MCell,^(^^12^^)^ and Smoldyn,^(^^13^^)^ but they are mainly dedicated to reaction-diffusion dynamics for specific biophysics applications. In particular, as mentioned in the review paper,^(^^14^^)^ they are “also known as Brownian motion simulators” and as such they hardly represent the diversity of particle motions observed in some applications.

The aim of particle-based models, as those exploited in the above references, is to represent the collective motion of particles and global biomolecule trafficking. The latter should ideally account for the stochastic displacement of all individual particles, but also for a possible regime switching of each trajectory, the time of appearance of new biomolecules and their lifetime. Moreover, interactions between biomolecules should be possible, for instance between different types of proteins, giving rise to the colocalization phenomena observed in several applications.^(^^15^^–^^18^^)^

Beyond the aforementioned popular softwares, there is already a vast variety of stochastic models introduced in the literature to represent the individual trajectories, allowing for instance for Brownian, confined, anomalous, or directed motions with variable velocities within the cell,^(^^7^^,^^8^^,^^19^^–^^26^^)^ or even supported along a cytoskeleton network.^(^^20^^,^^27^^,^^28^^)^ However, these dynamics are rarely prone to regime switching, though this feature is often observed in real applications.^(^^22^^)^ They also generally assume independence between particles. Regarding the time and location of appearance of new particles, the existing models (including those provided by the popular softwares) are unsophisticated if not ignoring this feature. A constant rate of birth is generally assumed and no interaction with the existing particles is considered for the location of appearance, ruling out any colocalized dynamics. The same restriction occurs for the dynamics of disappearance of particles. Consequently, there is still an avenue to improve the existing particle-based models in order to take into account this lack of features.

We propose in this contribution to leverage a tailored stochastic model introduced in Ref. (29), which is flexible enough to include all aforementioned features in an unified and theoretically well-grounded framework. In agreement with our objective, this so-called birth-death-move (BDM) spatial point process is a model for the dynamics of a system of particles, that move over time, while some new particles may appear in the cell and some existing particles may disappear. Moreover, each particle may be marked by a given label, for example, among different possible labeled proteins and/or different types of motion regimes, and this mark may change over time, for example, a particle may switch from one regime (e.g., Brownian) to another (e.g., directed motion). This switch of a mark is sometimes called a “mutation” in the literature, but we prefer here to use the term “transformation” to avoid misunderstanding with a genuine biological mutation. The trajectories can be driven by any continuous Markov diffusion model, that includes most models for individual trajectories previously considered in the literature, and some interactions may be introduced so that colocalization phenomena can be generated. The intensity of births, that govern the waiting time before the next appearance of a new particle, may depend on the current configuration of particles, and similarly for the intensity of deaths. For instance, we may design that the more biomolecules in the cell there are, the higher the death intensity is, implying a rapid disappearance. Some spatial effects may also be taken into account, in order to create distinct motion regimes in some regions of the cell, or to encourage some spatial regions for the appearance of a new particle, for example, nearby some existing particles due to colocalization.

In a nutshell, compared to existing particle-based stochastic models and softwares, our approach enables to simulate a vast variety of Markov trajectories for the system of particles, including interactions between them during their displacements, as well as in the dynamics of births and deaths, thus accounting for possible colocalization effects. Additionally, it allows for regime-switching within each individual trajectory.

**Figure 1.**
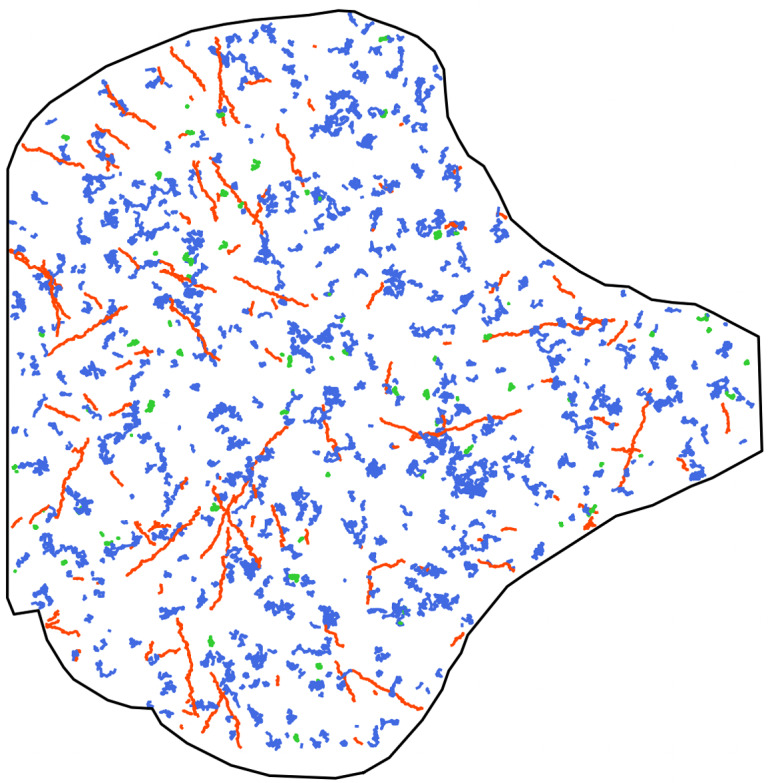
Left: set of all trajectories detected and tracked over a real-image sequence of Langerin proteins, colored by their estimated motion regime (Brownian in blue, directed motion in red, and confined motion in green). Right: result from a synthetic sequence generated by our stochastic model.

The remainder of the article is organized as follows. In Section 2, we give the precise definition of our stochastic process, and we in particular list all ingredients needed to fully specify the model. An iterative construction is presented in Section 2.2, clarifying how the dynamics proceeds, and an effective simulation algorithm is formally detailed in the Appendix and made available online. In Section 2.3, we provide numerous examples for the specifications of the model, that we think are relevant for many real biomolecules dynamics. In Section 3, we demonstrate the potential of our approach by focusing on the joint dynamics of Langerin and Rab11 proteins being involved in membrane trafficking. We start by the inspection of a real dataset in order to calibrate judiciously the different parameters of the BDM model to be in agreement with this example. The dataset consists of a sequence of images acquired by 3D multi-angle TIRF (total internal reflection fluorescence) microscopy technique,^(^^30^^)^ depicting the locations of Langerin and Rab11 proteins close to the plasma membrane of the cell, specifically over a distance of 1 μm in the *z*-axis. After some post-processing, the sequence shows a set of trajectories for both type of proteins, that follow different motion regimes, are spatially distributed within the cell in a specific way, and occur at different periods during the sequence, which is in perfect line with the dynamics of a BDM process. The observed trajectories for the Langerin channel are depicted on the leftmost plot of Figure 1. We compute a set of descriptors from this dataset in order to calibrate the parameters of our stochastic process, but also to create some benchmark features for the assessment of our synthetic sequences. Finally, in Section 3.2, we generate several simulated sequences and show that they exhibit comparable features as those observed in the real sequence. An example of generated trajectories is displayed in the rightmost plot of Figure 1. For this illustration, although the individual trajectories exhibit basic dynamics (they are independent, homogeneous in space and either follow Brownian, confined, or directed motions), the advantage of our approach lies in its ability to incorporate regime switching within these trajectories and to account for the colocalization phenomenon when new particles appear.

Supplementary materials, including the Python code for simulation, the raw data, and some further simulated sequences, are available in our online GitHub repository at https://github.com/balsollier-lisa/BDM-generator-for-bioimaging.

## 2. The mathematical model

### 2.1. Heuristic and notations

In order to mimic the dynamics of biomolecules, we consider a multitype BDM process with mutations, denoted by 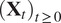 . This process is a generalization of BDM processes, as introduced in Ref. (29). In the following, to avoid misunderstanding we rather use the term “transformation” instead of “mutation,” as explained in Section 1. This section describes the spatiotemporal dynamics of 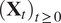 and introduces some notation.

At each time 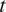 , 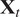 is a collection of particles located in a bounded set 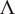 of 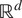 . Each particle is assigned a mark that represents a certain feature. We denote by 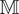 the collection of possible marks. Through time, the particles move (possibly depending on their associated mark and in interaction with each other) and three sudden changes may occur, that we call “jumps”:

1. a “birth”: a new particle, assigned with a mark, may appear;
2. a “death”: an existing particle may disappear;
3. a “transformation”: the mark of an existing particle may change.

*Example*: In our biological application treated in Section 3, 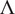 represents a cell in dimension 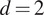 or 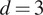 . We observe inside this cell two types of particles, associated to Langerin and Rab11 proteins, and each of them moves according to three different possible regimes: Brownian, directed, or confined motion. For this example, each particle is therefore marked out of six possibilities, whether it is associated to Langerin (L) or Rab11 (R), and depending on its motion regime (1 to 3), so that 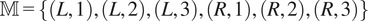 . Through time, each particle moves independently of the others according to its motion regime and eventually a new particle may appear, an existing one may disappear, and the motion regime of some particles may change.

We denote by 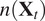 the number of particles at time 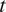 . Each particle 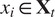 , for 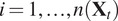 , is decomposed as 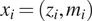 where 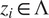 stands for its position while 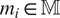 denotes its mark. Accordingly we have 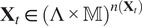 . (Strictly speaking the ordering of particles in 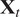 does not matter, because any permutation of particles leads to the same collection of particles. We choose in this article to bypass this nuance and use the same notation as if 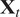 was a vector a particles, even it is actually a set of particles.) Since the number of particles changes over time, the stochastic process 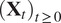 takes its values in the space

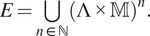

To stress the fact that 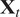 is not a simple value but encodes the positions and marks of a system of particles, we will say that this system at time 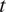 is in *configuration* 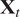 .

To fully specify the dynamics of 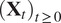 , we need the following ingredients:

1. A system of equations 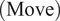 that rules the way each particle of 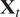 moves continuously between two jumps. We will typically consider a system of stochastic differential equations acting on the position of each particle, possibly depending on their associated mark and in interaction with the other particles;
2. Three continuous bounded functions 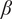 , 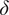 , and 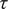 from 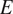 to 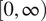 , called birth, death and transformation intensity functions respectively, that govern the waiting times before a new birth, a new death, and a new transformation. At each time 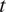 , we may interpret 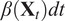 as the probability that a birth occurs in the interval 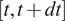 , given that the system of particles is in the configuration 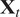 , and similarly for 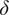 and 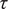 .
3. Three transition probability functions that indicate how each jump occurs:

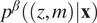
 : probability density function that the birth occurs at the position 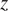 with the mark 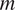 , given that there is a birth and that the system of particles is in configuration 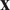 at the birth time;

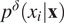
 , for 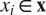 : probability that the death concerns the particle 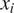 in 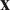 , given that there is a death and that the system of particles is in configuration 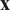 at the death time;

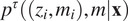
 , for 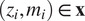 : probability that the particle 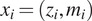 in 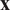 changes its mark and that this transformation leads to the new mark 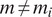 , given that there is a transformation and that the system of particles is in configuration 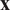 at the transformation time.

We provide in Section 2.3 some examples for the choice of these characteristics. Finally, we will denote by 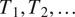 the jump times of the process and we agree that 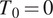 .

### 2.2. Algorithmic construction

Assume we are given the characteristics of the process 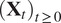 as introduced in the previous section, that are the system of equations 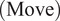 , the intensity functions 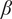 , 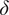 , 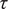 , and the transition probability functions 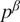 , 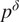 , and 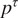 . Then, starting from an initial configuration 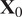 at time 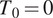 , we construct iteratively the process in the time interval 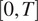 as follows. Here we set 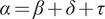 to be the total intensity of jumps.

1. Generate 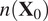 continuous trajectories as solutions of 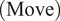 in the interval 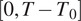 , given the initial conditions 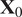 . Denote 
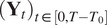
 these trajectories.
2. By flipping a coin, test whether the jump time 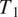 occurs after 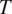 (this is with probability 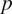 ) or before 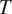 (this is with probability 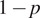 ), where

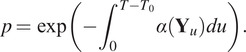

- If 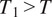 , then 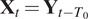 for all 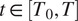 , which completes the simulation.
- Otherwise, we continue by generating 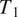 in 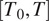 and the associated jump as in the following.
  3. Generate 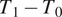 , given that 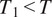 , according to the probability distribution

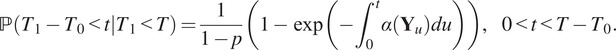



The process until the time 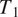 is then given by the generated trajectories, that is,

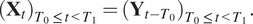

4. Draw which kind of jump occurs at 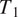 (we denote by 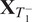 the configuration of the process just before the jump, which is 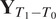 by continuity of 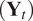 ):

- this is a birth with probability 
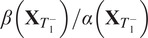
 ;
- this is a death with probability 
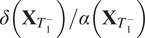
 ;
- this is a transformation with probability 
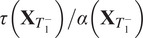
 .
  5. Generate the jump at 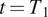 to get 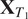 as follows:
- if this is a birth, generate the new particle 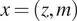 according to the probability density function 
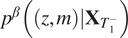
 . Then set 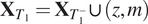 ;
- if this is a death, draw which particle 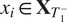 to delete according to the probability 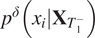 , for 
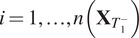
 . Then set 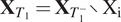 ;
- if this is a transformation, draw which particle 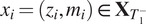 is transformed and generate its transformation according to 
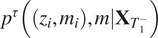
 , for 
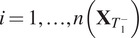
 . Then set 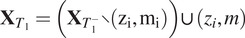 .
  6. Back to step 1 with 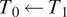 and 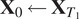 in order to generate the new trajectories starting from 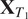 and the next jump time 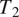 , and so on.

In the first step of the above construction, the trajectories are generated up to the final time 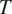 . It is however very likely that the next jump occurs much before 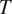 so that it would be sufficient and computationally more efficient to generate these trajectories on a shorter time interval. We provide in the Appendix a formal algorithm of simulation of 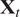 for 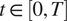 , following the above construction and including the latter idea. This algorithm has been implemented in Python and is available in our GitHub repository.

From a theoretical side, note that the specific exponential form of the probability distribution of the inter-jump waiting time in step 3 is necessary to imply the interpretation of 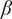 , 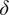 , and 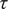 explained in the previous section. This exponential form also implies that 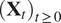 is a Markov process, meaning that its future dynamics only depends on its present configuration. We refer to Ref. (29) for more details about these theoretical aspects.

### 2.3. Exemplified specifications of the model

#### 2.3.1. The inter-jumps motion

Recall that during an inter-jump period, the process 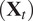 has a constant cardinality 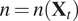 and the marks of all its particles remain constant. We denote by 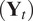 a system of 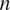 such particles 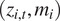 , for 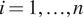 , where 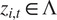 represents the position of the 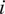 th particle at time 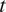 and 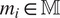 is its constant mark, that is

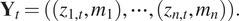

In agreement with the construction of the previous section, the inter-jump trajectory of each particle of 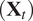 will coincide with the 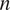 trajectories of 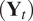 during this period.

As a general example, we assume that 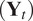 follows the following system of stochastic differential equation, starting at 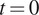 at the configuration 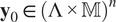 ,

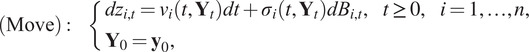

where the drift functions 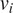 take their values in 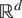 , the diffusion 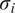 are nonnegative functions, and 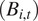 , 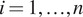 , are 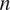 independent standard Brownian motions in 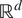 . Here, 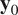 , 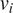 , and 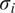 are free parameters to be chosen.

Some conditions on the drift and diffusion functions are necessary to ensure the existence and unicity of the solution of 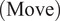 . This holds for instance if these functions are Lipschitz,^(^^31^^)^ a condition met for the following examples. In addition, since each particle is supposed to evolve in the bounded set 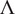 of 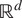 , we need in practice to force the trajectories of 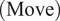 to stay in 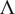 . This may be achieved by reflecting the trajectories at the boundary of 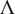 .

In its general form, 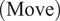 allows the motion of each particle to depend on its mark, but also on the position and mark of the other particles (that are part of 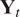 ). We detail several examples below, that may be realistic for biological applications.

Example 1 *(Brownian motions)*: If 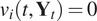 and 
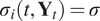
 (for 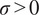 ) is constant, then each particle follows a Brownian motion with the same diffusion coefficient 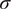 , independently of the other particles.

Example 2 *(spatially varying diffusion coefficients)*: If 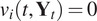 and 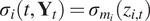 , where 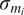 is a positive function defined on 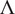 , then each particle follows an independent diffusive motion, where the diffusive coefficient depends on the associated mark and may vary in space. For instance, assume that 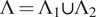 with 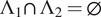 and that for 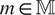 ,

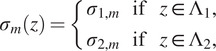

where 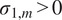 , 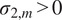 . Then each particle with mark 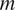 follows locally in 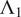 a Brownian motion with diffusion coefficient 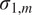 and locally in 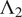 a Brownian motion with diffusion coefficient 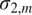 . Note that as such, 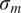 is not Lipschitz and it needs to be smooth so as to fit the theoretical setting. This may be achieved by taking the convolution of 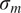 by a bump function.

Example 3 *(directed and confined motions)*: If 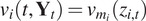 and 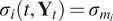 , where 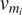 is defined on 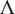 and 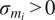 , then each particle evolves independently of each other with a drift and a diffusion coefficient that depend on its mark. This example includes the directed motion considered in Ref. (22) when 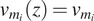 is a constant drift. It also includes the Ornstein–Uhlenbeck dynamics, also considered in Ref. (22), when 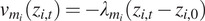 , where 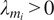 can be interpreted as a force of attraction toward the initial position 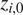 , leading to a confined trajectory.

Example 4 *(interacting particles)*: In this example, we show how we can include interactions between the particles through a Langevin dynamics. To do so, we introduce, for 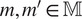 , pairwise interaction functions 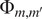 , as considered in statistical physics: For 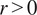 , 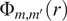 represents the pairwise interaction between a particle with mark 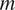 and a particle with mark 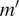 at a distance 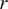 apart. If 
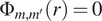
 , there is no interaction, if 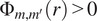 there is inhibition between the two particles at distance 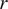 , and if 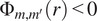 there is attraction. Examples of inhibitive interaction functions can be found in Ref. (32). The (overdamped) Langevin dynamics associated to these interactions reads as 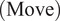 with 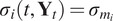 , 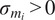 , and

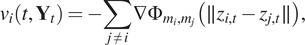

where 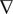 denotes the Gradient operator. Accordingly, each particle moves in a direction that tend to decrease the value of the pairwise interaction function with the other particles.

Example 5 *(colocalized particles)*: Assume that some particles, say with mark 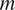 , are thought to be colocalized with particles having the mark 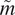 . This means that we expect the former to be localized nearby the latter and to follow approximately the same motion. Specifically, to let the particle 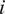 with mark 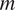 be colocalized with the particle 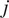 with mark 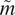 , we may simply define 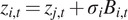 , 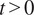 , where 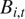 is a standard Brownian motion in 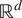 representing the deviation of the trajectory 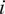 around the trajectory 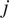 , and 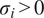 quantifies the strength of this deviation. Here 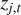 may be defined as in the previous examples, for instance as the typical trajectory of a particle with mark 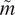 .

#### 2.3.2. The intensity functions

Recall that the intensity functions 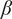 , 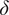 , and 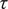 rule the waiting times until the next birth, death, and transformation, respectively. Heuristically, the probability that a birth occurs in the time interval 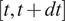 given that the particles are in configuration 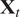 is 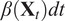 , and similarly for 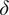 and 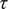 . As a consequence these probabilities may evolve over time according to the configuration of particles, making for instance a death more likely to happen when there are many particles or a high concentration of them in some region, due to competition. We provide some natural examples below. For each example, any of 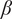 , 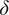 , or 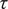 can be set similarly, even if we focus only on one of them.

Example 6 *(constant intensities)*: The simplest situation is when the intensity functions are constant, for instance 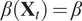 with 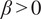 . Then births appear at a constant rate and we can expect that in average 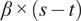 new particles appear during the interval 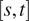 .

Example 7 *(intensities depending on the cardinality)*: If 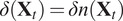 , with 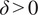 , then the more particles there are, the more deaths we observe. This is a natural situation when each particle is thought to have a constant death rate 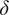 , so that the total death intensity for the system of particles at time 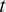 is just the sum of them, that is 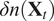 .

Example 8 *(spatially varying intensities)*: Assume that the mark of a particle (say its motion regime) has more chance to change in some region of 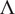 than another, then the transformation intensity 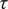 may reflect this dependency. Let for instance 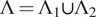 with 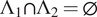 and define 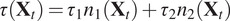 where 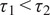 and 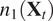 (resp. 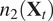 ) denotes the number of particles in 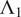 (resp. in 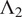 ). Then for a given cardinality 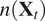 , the more proportion of particles in 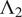 , the more transformations happen. Note that in order to be rigorous, we should consider a continuous version of 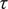 , which can be achieved by convolution with a bump function.

Example 9 *(transformation due to colocalization)*: Assume that some 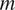 -particles (that are the particles with mark 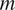 ) can be colocalized with some 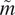 -particles. Assume in addition that the particles are assigned a second mark that encodes their motion regime (e.g., diffuse, confined, or directed). Eventually, during the dynamics of particles, a noncolocolized 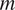 -particle may become colocolized with a 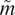 -particle, meaning that it becomes 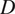 -close to a 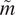 -particle, where 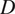 is some prescribed colocalization distance. If so, we may expect that the motion regime of the 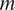 -particle becomes similar as the 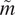 -particle, so that a transformation must occur. Let 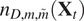 be the number of 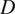 -close pairs of particles with marks 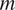 and 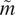 , whose motion regimes are different. Then we may define 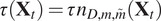 , for some 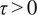 , so that a transformation (of motion regime here) is very likely to occur when the aforementioned situation happen. Note that if 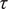 is large, such transformation will quickly happen as soon as 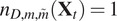 , and so 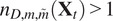 will be unlikely to be observed. Here again, a smooth version of 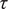 can be introduced by convolution to ensure its continuity.

#### 2.3.3. The transition probability functions

We detail examples for the three possible transitions, in order below: births, deaths, and transformations.

For the births, remember that 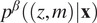 denotes the probability density function (pdf) that a particle appears at the position 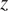 with the mark 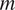 , given that the system of particles are in configuration 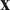 . To set this probability, two approaches are possible:

1. First drawing the mark 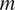 of the new particle with respect to some probability 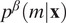 , then the position of the new particle given its mark according to some pdf 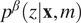 . This leads to the decomposition 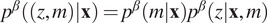 .
2. First generating the position of the new particle with respect to some pdf 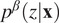 , then its mark 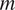 given the position with probability 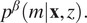 This leads to the decomposition 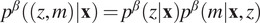 .

Example 10 *(uniform births)*: This is the simple example where the births do not depend on the environment, are uniform in space and the marks are drawn with respect to some prescribed probabilities 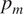 , where 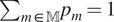 . The two above approaches then coincide with 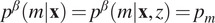 and 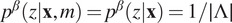 for 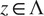 .

Example 11 *(colocalized births)*: We adopt here the first approach above. We first draw the marks independently of the environment by setting 
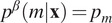
 with 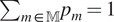 , as in the previous example. Second, in order to generate the position of a new 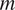 -particle, thought to be colocalized with the 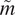 -particles, we may use a mixture of isotropic normal distribution, centered at each 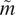 -particle, with deviation 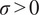 . Denoting by 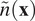 the number of 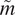 -particles in 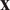 and 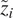 their positions ( 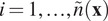 ), this means that(1)

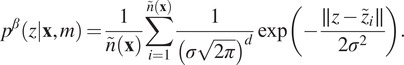

Note that to be rigorous 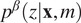 should be restricted to 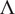 with a proper normalization, otherwise some particles might be generated outside 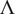 . We omit these details.

Example 12 *(spatially dependent new marks)*: We may adopt the second approach by first generating a uniform position for the new particle, that is, 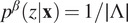 for 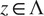 , and second by drawing the mark according to the generated position. Let for instance 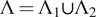 with 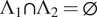 and set

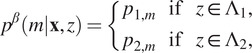

where 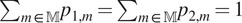 . Then depending on the position, the distribution of the marks may be different.

We now focus on the death transition, namely the probability 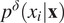 , for 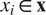 , that the particle 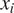 in 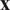 disappears when there is a death.

Example 13 *(uniform deaths)*: The simplest example is when a death occurs uniformly over the existing particles, that is 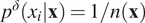 for 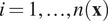 .

Example 14 *(deaths due to competition)*: We may imagine that, due to competition, a particle is more likely to disappear if there are too many neighbors around it. Let 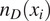 be the number of neighboring particles around 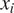 within distance 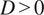 . To take into account the competition at distance 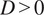 , we may define 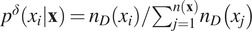 . Similarly, if relevant, we may count the number of neighbors of a certain mark only.

Finally, we focus on 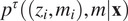 , for 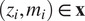 , which is the probability that the particle 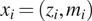 in 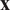 changes its mark from 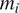 to 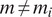 , when a transformation happens. Similarly, as for the birth transition probability, it is natural to decompose this probability as

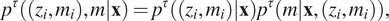

where 
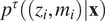
 represents the probability to choose the particle 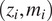 in the configuration 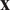 , in order to change its mark, and 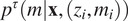 is the probability to choose the new mark 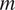 given that the transformed particle is located at 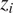 with mark 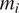 .

Example 15 *(transformations independent on the environment)*: A typical situation is when the particle to transform is drawn uniformly over the existing particles, that is 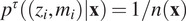 , and the transformation is carried out independently on the environment, according to a transition matrix with entries 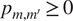 , 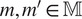 , representing the probability to be transformed from mark 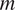 to mark 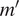 . Here, for all 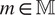 , we assume 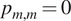 in order to ensure a genuine transformation, and of course 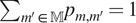 . With this formalism, we thus have 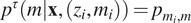 .

Example 16 *(spatially dependent transformations)*: To make the previous example spatially dependent, introduce 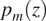 , a pdf in 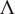 representing the locations in 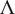 where a particle with mark 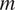 is more or less likely to be transformed. Then we may set

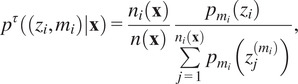

where 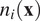 denotes the number of particles with marks 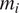 in 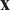 and 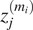 , 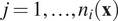 , their positions. In this expression 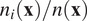 is a weight accounting for the prevalence of mark 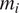 in 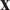 and the sum in the denominator is a normalization so that the probabilities sum to 1. Note that if 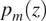 is the uniform pdf on 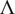 , then we recover the uniform distribution 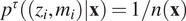 . Furthermore, once the particle is chosen as above, we may apply a spatially dependent transformation as follows. Let 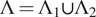 with 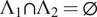 and let two different transition matrices with respective entries 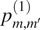 and 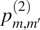 , for 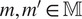 . Then we may set

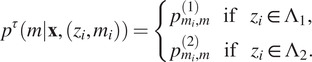

Accordingly, the transformation does not follow the same distribution, whether the chosen particle to be transformed is located in 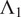 or 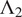 .

Example 17 *(transformation due to colocalization)*: Assume that we are in the same situation as in Example 9 where 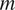 -particles can be colocalized to 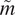 -particles. We assume like in this example that a transformation occurs if 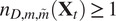 , where 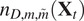 denotes the number of 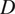 -close pairs of particles with marks 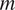 and 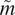 , whose motion regimes are different. Then, when a transformation happens, we may choose the 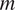 -particle to be transformed uniformly over those 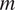 -particles that are 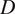 -close to a 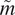 -particle with a different motion regime. Then the transformation makes the motion regime of the selected 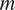 -particle similar as the motion regime of its closest 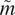 -particle.

## 3. Application to the joint dynamics of Langerin/Rab11 proteins

### 3.1. Description of the dataset

**Figure 2.**
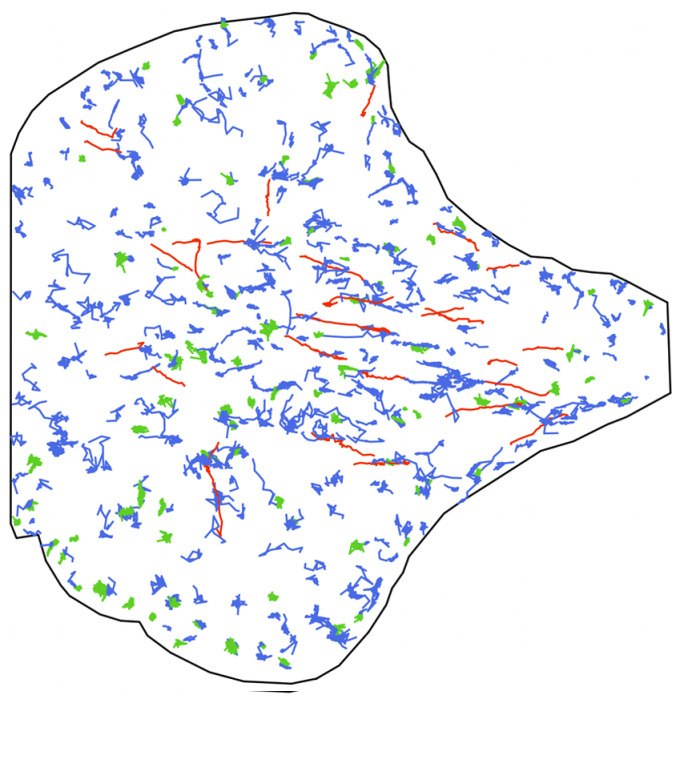
(a) First frame of the raw sequence showing in bright spots the location of Langerin proteins; (b) same as (a) for the Rab11 proteins; (c) set of all trajectories detected and tracked over the sequence of Langerin proteins colored by their estimated motion regime (Brownian in blue, directed motion in red and confined motion in green); (d) same as (c) for the Rab11 proteins.

The dataset we consider comes from the observation by a 3D multi-angle TIRF (total internal reflection fluorescence) microscopy technique of the intracellular trafficking of YFP Langerin and m-Cherry Rab11 proteins in a RPE1 living cell,^(^^30^^)^ specifically projected along the *z*-axis onto the 2D plane close to the plasma membrane. This provides a 2D image sequence of 1199 frames, each lasting 140 ms and showing the simultaneous locations of the two types of proteins. The two images at the top of Figure 2 depict the first frame of the raw sequence for the Langerin fluorescent channel and the Rab11 fluorescent channel, respectively, recorded simultaneously using a dual-view optical device. Note that the cell adheres on a fibronectin micropattern, which constrains intracellular constituents such as cytoskeleton elements and gives a reproducible shape, explaining the “umbrella” shape of the cell. These raw sequences are post-processed following Refs. (33, 34), then each bright spot is represented by a single point, and we apply the U-track algorithm^(^^35^^)^ to estimate particle trajectories. The bottom images of Figure 2 show the resulting trajectories for the Langerin channel and the Rab11 channel, respectively. These trajectories have been further analyzed by the method developed in Ref. (22) to classify them into three diffusion regimes: Brownian, directed, and confined, which corresponds to the blue, red, and green colors, respectively, in Figure 2.

To be more specific in the analysis of all trajectories, we fit three parametric models to each of them, following Ref. (22), depending on their regime:

1. for a Brownian regime (in blue): a Brownian motion,
2. for a directed motion regime (in red): a Brownian motion with constant drift,
3. for a confined motion regime (in green): an Ornstein–Uhlenbeck process.

Each trajectory has its individual parameters (see Examples 1 and 3), estimated by maximum likelihood.^(^^36^^)^ Furthermore, some trajectories may change from one regime to another, which corresponds to a “transformation” in the BDM model that will be specified in the next section.

**Figure 3.**
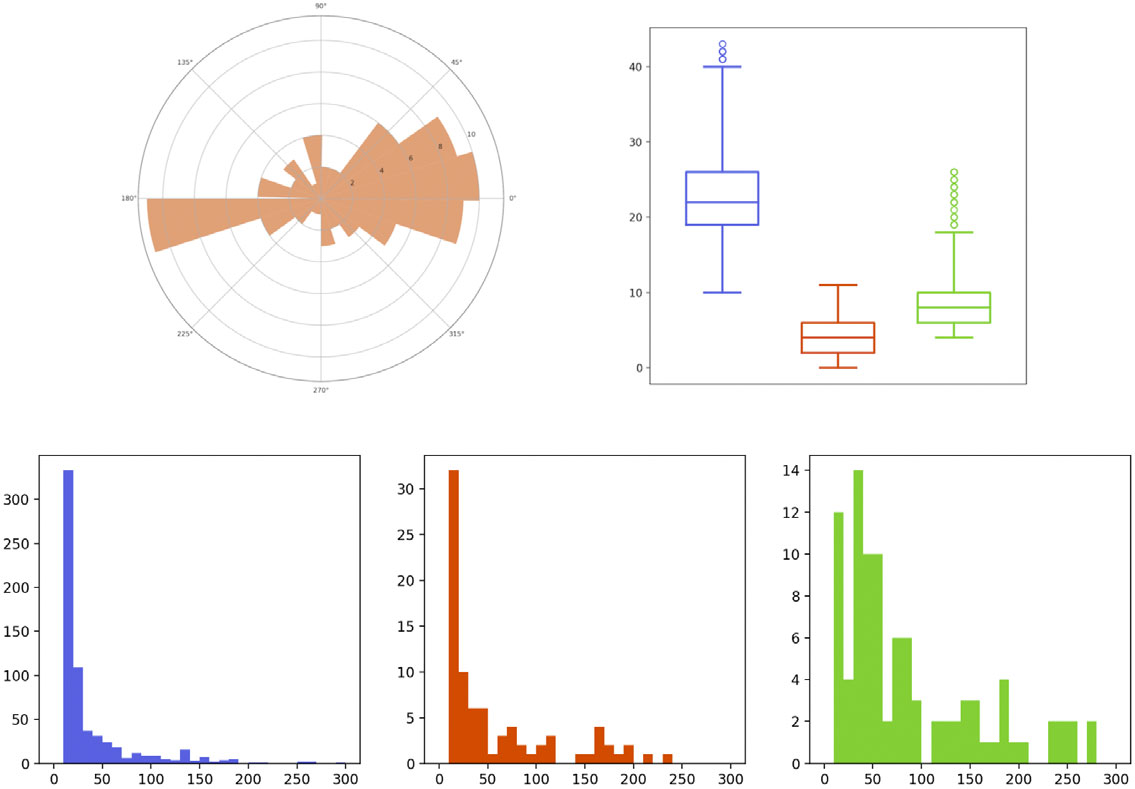
Descriptors of the Langerin trajectories of the real-data sequence. Top-left: circular histogram of the deviation angle (from the direction toward the center of the cell) of the drifts of the directed trajectories. Top-right: boxplots of the number of trajectories per frame, according to their regime (blue: Brownian, red: directed motion, green: confined motion). Bottom: histograms of the lifetime (in frames) of each trajectory according to its regime (same color label).

Figure 3 summarizes different features of the obtained trajectories for the Langerin sequence (the same characteristics have been analyzed for the Rab11 sequence, but are not detailed here). The histograms at the bottom display the duration of all trajectories (in frames), according to their regime. We can observe that the (blue) Brownian and (red) directed trajectories have quite a short lifetime in average, in comparison with the confined trajectories (in green). The top-right boxplots represent the distribution of the number of particles per frame, according to their regime: there is a majority of Brownian motions, followed by confined motions and a minority of directed motions. Finally, the top-left circular histogram aims at depicting the orientation of the drift vectors for the directed (red) trajectories. Specifically, for this plot, we have recorded the deviation of the drift angle (in degrees) with respect to the direction toward the center of the cell. For instance, this deviation is 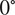 if the drift goes toward the center, and 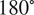 if it goes in the opposite direction. It appears from this plot that most deviation angles are around 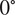 or 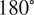 , meaning that the red trajectories mainly move in a radial direction going to (or starting from) the center of the cell.

The above descriptors will be helpful to calibrate the parameters of the BDM model in the next section and they will also serve as benchmarks to evaluate the quality of our simulations. However, it is important to keep in mind that they come with some approximations and errors induced by imperfect tracking algorithms. In particular, no trajectory can last less than 10 frames in the data, which is a minimal length of detection for our tracking method. It is also clear in the bottom plots of Figure 1, that some directed trajectories appear wrongly in blue, which can be explained by the multiple testing procedure of Ref. (22) that aims at minimizing the number of false positives (that are bad green or bad red trajectories) to the detriment of possibly too many false negatives (that are wrong blue trajectories).

Concerning the births and deaths of trajectories, we summarize in Table 1 their total numbers observed in the real dataset, according to the type of proteins and motion regime. The number of regime transformations is in turn given in Table 2 for the Langerin proteins. For the Rab11 proteins, only one switching from a Brownian motion to a confined motion was observed during the sequence.

**Table 1.** Total number of births and deaths of trajectories observed in the realdataset sequence, according to the type of proteins and the motion regime

		Brownian	Directed	Confined	Total	
Births	Langerin	603	78	66	747	1248
	Rab11	393	24	84	501	
Deaths	Langerin	602	77	89	768	1282
	Rab11	395	26	93	514

**Table 2.** Total number of regime transformations observed in the realdataset sequence of Langerin trajectories

From/To	Brownian	Directed	Confined
Brownian	0	0	9
Directed	0	0	1
Confined	5	0	0

To address in detail these jumps dynamics, we leverage the study carried out for the same dataset in Ref. (29), where it has been concluded that for each type of proteins and motion regimes, the birth intensity is constant, like in Example 6, while the death intensity is proportional to the number of existing particles, like in Example 7. Given the small number of observed motion regime transformations, its intensity can also be considered as constant. Concerning the transition probability functions, the deaths occur uniformly over all existing particles, like in Example 13. As to the birth transition, there is no reason to choose another density than the uniform distribution over the cell for the Rab11 proteins (Example 10). But due to colocalization (as observed for this dataset in Ref. (18)), the birth density for the Langerin positive structures can be approximated by a mixture between a uniform distribution, for 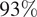 of the Langerin births, and a colocalized density around the existing Rab11 vesicles, like in Example 11, for 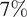 of the Langerin births. These proportions, along with the other parameters, have been estimated by maximum likelihood, the theoretical foundations of which can be found in Refs. (37, 38) for stochastic models that include the BDM model. Note however that at this step, the goal is to provide a guideline to set the parameters of the BDM model in order to generate realistic realizations, as carried out in the next section. For this reason, any alternative estimation method or biological expertizes to set the parameters could be appropriate.

### 3.2. Simulation of synthetic sequences

#### 3.2.1. Model parameters setting

Based on the data analysis of the previous section, we are now in position to specify all characteristics of the BDM process with transformations presented in Section 2, so as to mimic the joint dynamics of Langerin/Rab11 proteins within a cell. To make the connection with the theoretical notation, the region of interest 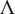 represents the cell in dimension 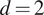 . Each particle in 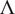 will be marked by a label from the set 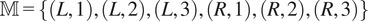 , where 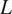 stands for the Langerin proteins, 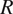 for the Rab11 proteins, and the number 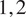 , or 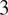 indicates the motion regime of the particle: Brownian, directed, or confined, respectively.

Concerning the motion of each trajectory, it follows the regime indicated by its mark and is in agreement with the observed trajectories from the real dataset detailed in the previous section, see also Examples 1 and 3:

1. For a Brownian motion, we draw the diffusion coefficient according to the empirical distribution of the diffusion coefficients estimated from the Brownian motions of the real dataset, for the same type of proteins ( 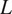 or 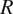 );
2. For a directed motion, we generate a Brownian motion with constant drift, with the same strategy for the choice of the diffusion coefficient, and where the drift vector is chosen as follows: it is by default oriented toward the center of the cell, this orientation being subjected to a deviation drawn from the empirical distribution depicted in the top-left circular histogram of Figure 3. In addition, its norm is drawn from the empirical distribution of the drift norms observed from the real dataset. Here again, each set of parameters is distinct for the Langerin and Rab11 proteins;
3. For a confined motion, we generate an Ornstein–Uhlenbeck process with diffusion coefficient 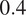 for all particles (which is the average from the real dataset), and parameter 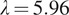 for the Langerin proteins, and 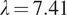 for the Rab11 proteins.

In these values, the unit is pixels, and one pixel is 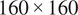 nm 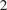 in our images.

Concerning the intensity functions, we set the birth intensity and the transformation intensity to constant values, as concluded from the real-data analysis. In agreement with Table 1, the total birth intensity can be estimated by 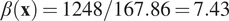 , whatever the configuration 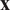 of particles is, because 1248 is the total number of observed births and 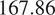 is the total time length of the sequence (in seconds). Similarly, we set 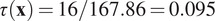 since 16 transformations have been observed in the real sequence. For the death intensity, for each mark 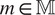 , we let it proportional to the number of particles, that is 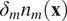 , where 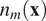 is the number of particles with mark 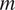 in the configuration 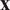 and 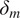 has been estimated from the real-dataset as follows: 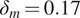 if 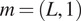 , 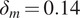 if 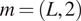 , 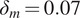 if 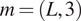 , 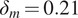 if 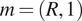 , 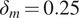 if 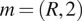 , and 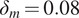 if 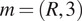 . The total death intensity for the configuration 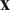 of particles is then 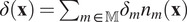 .

Finally, we set the transition probability functions as follows. For the death transition, the probability to kill the particle 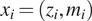 in the configuration 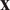 is set to

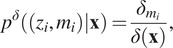

which means that we first draw the mark 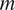 with probability 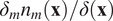 and then the particle uniformly among all existing particles with mark 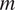 . For the transformation transition, we first select the type of proteins to transform with probability 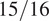 for Langerin and 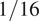 for Rab11, in line with the transformations rates observed in the real sequence, second we choose a particle uniformly among all existing particles of this type, and third, as in Example 15, we apply a regime transformation with respect to the following transition matrix (from the regime in rows to the regime in columns):

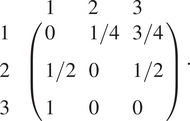



This matrix is in agreement with Table 2 concerning the Langerin proteins, where we have added some possible transitions from regime 1 to 2, and from regime 2 to 1, that appear to us likely to occur, even if they were not observed in the (quite rare) transformations in the real-sequence. The same transition matrix has been set for the Rab11 proteins, since there is not enough observed transformations in the real sequence (only one) to design a finer choice.

It remains to set the birth transition probability. First, we select which type of protein to create: following Table 1, it is a Langerin protein with probability 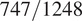 and a Rab11 protein with probability 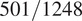 . If the selected type is Rab11, then it is generated uniformly in the cell with regime 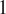 with probability 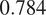 , 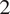 with probability 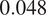 , and 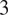 with probability 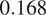 , which corresponds to the relative proportion of births of each regime over all births for the real Rab11 sequence. If the selected type is Langerin, then we flip a coin for colocalization with probability 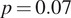 . If there is no colocalization, then the new Langerin protein is generated uniformly in the cell with regime 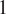 with probability 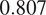 , 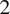 with probability 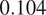 , and 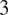 with probability 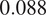 (the observed relative proportions of births). If there is colocalization, then the new Langerin protein is generated around an existing Rab11 protein according to the density (1) in Example 11, where by maximum likelihood estimation 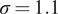 . In this case, the regime of the new Langerin protein and its drift vector for a directed motion are similar as those of its colocalized Rab11 protein.

#### 3.2.2. Analysis of resulting simulations

**Figure 4.**
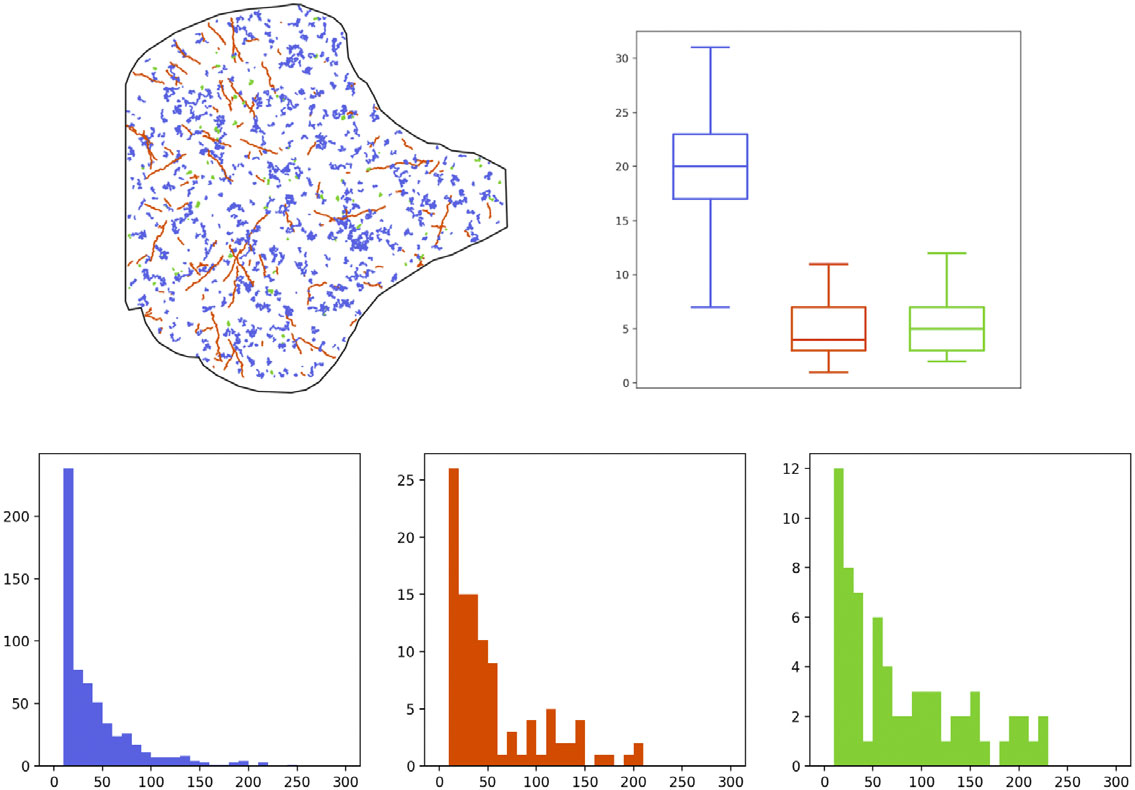
Descriptors of the Langerin trajectories of a first simulated sequence. Top-left: set of trajectories, colored according to their motion regime (blue: Brownian, red: directed, green: confined). Top-right: boxplots of the number of trajectories per frame, according to their regime (same color label). Bottom: histograms of the lifetime (in frames) of each trajectory according to its regime (same color label).

**Figure 5.**
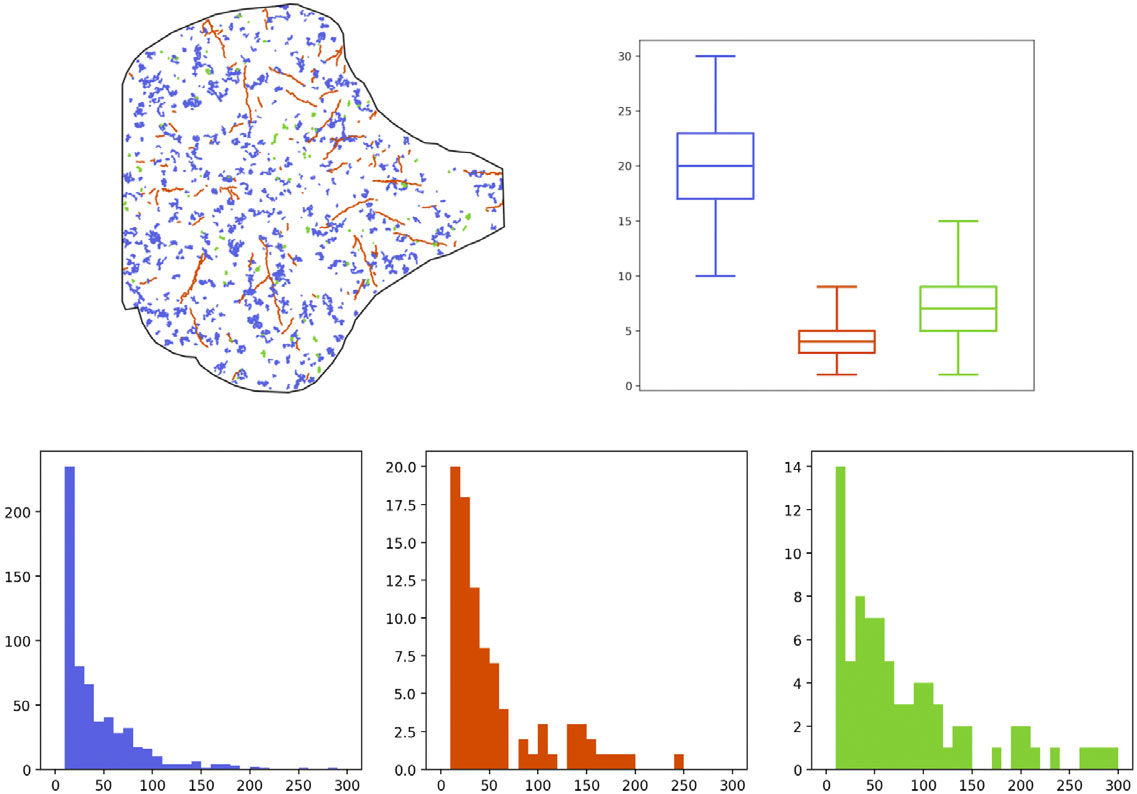
Descriptors of the Langerin trajectories of a second simulated sequence, as in Figure 4.

We have generated 100 sequences following the model of the previous section, during the same time length as the real sequence of Section 3.1, that is 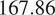 s for 1199 frames. Some descriptors concerning the generated Langerin trajectories coming from two simulated sequences are depicted in Figures 4 and 5, that are to be compared with the similar outputs of the real data in Figures 2 and 3. The results for other simulated sequences can be seen in our GitHub repository. We have also summarized the mean number of births and deaths over the 100 simulated sequences in Table 3, to be compared with Table 1. Both graphical and quantitative results demonstrate that our model is able to create a joint dynamics with comparable features as those observed in the real-data sequence.

**Table 3.** Mean total number of births and deaths of trajectories per sequence, over 100 simulated sequences

		Brownian	Directed	Confined	Total	
Birth	Langerin	581	85	76	742	1239
	Rab11	391	22	83	496	
Death	Langerin	584	86	77	747	1248
	Rab11	397	23	80	500	

## Data Availability

The real data presented in the manuscript and replication code may be obtained from the authors and can be found in our GitHub repository at https://github.com/balsollier-lisa/BDM-generator-for-bioimaging.

## A. Appendix

### A.1. Algorithm for simulation

We provide in this appendix a formal algorithm to simulate a BDM process with mutations (or transformations), following the construction of Section 2.2. Its implementation is available in our GitHub repository at https://github.com/balsollier-lisa/BDM-generator-for-bioimaging. It is a refinement of the algorithm introduced in Ref. (29) for a BDM process (without transformations). The idea is to generate the inter-jump move on a small time length  , then to test whether a jump has occurred during this period (this is with probability  in the following Algorithm 1). If so, we generate the jump time and the jump. If not, we continue the simulation of the inter-jump move on a further time length  , test whether a jump has occurred, and so on. The algorithm is valid whatever  is, but an efficient choice is to set a small value for  . A default recommendation is to set  as the discretization step used to simulate the trajectories in the  algorithm (which is an input of our algorithm, see below).

We let as in Section 2.2  and  . We denote in Algorithm 2  the configuration just before the jump time  . In order to run Algorithms 1 and 2, we need the following inputs:

1. : final time of simulation;
2. : initial configuration of particles;
3. : small time length for piecewise simulation;
4. ,  ,  : intensity functions of births, deaths, and transformations;
5. ,  ,  : transition probability for a birth, a death, and a transformation;
6. : algorithm that returns, for  and  ,  (discretized) trajectories on  following the system of SDEs  with initial configuration  .

Algorithm 1. Simulation on the time interval  
Algorithm 2. Simulation of the jump at  given

### A.2. Conclusion and perspectives

We have leveraged an original stochastic model, namely a multitype BDM process with transformations, in order to simulate biomolecules dynamics within a cell. This stochastic process not only models the individual trajectory of particles with any Markovian dynamics, but it is also able to generate the appearance (i.e., birth), disappearance (i.e., death), and regime switching (i.e., transformation) of each trajectory over time. Importantly, interactions between particles can be included, accounting for the possible colocalization phenomenon. The model is very flexible and is specified thanks to three sets of parameters: (1) a system describing the set of trajectories (typically a system of stochastic differential equations); (2) the intensity functions, ruling the waiting time before a new appearance, a disappearance or a switching; (3) the transition probability functions, driving where a new particle appears when there is a birth, which particle disappears when there is a death, and which particle switches its regime (and how) when there is a transformation. Numerous examples of these model specifications have been detailed. As an illustration, we demonstrated the relevance of this approach by generating the joint dynamics of Langerin/Rab11 proteins within a cell, based on a preliminary data-based analysis in order to finely calibrate the model.

Since the model is very flexible, an important step is the choice of model characteristics and parameters. The calibration carried out for our illustration is specific to the application at hand, and of course another calibration must be carried out for another application. In our case, we used one observed sequence of Langerin/Rab11 proteins. In order to improve the choice of parameters, a deeper empirical study based from several image sequences might help calibrating robustly the model. Once the parameters are fitted, the simulation of a sequence is quite fast: about one minute on an regular laptop for the generation of 2000 frames containing each about 70 trajectories in interaction. In general terms, the bottleneck is the simulation of all trajectories between two jumps: if each particle moves independently of the others, this scales linearly with the number of particles and parallelization is easy to set up. When complicated interactions are introduced between particles, then the simulation of all trajectories scales badly with the number of particles. As a restriction, due to the Markovian framework ensuring the theoretical well-posedness of the model, anomalous trajectories^(^^19^^,^^25^^,^^39^^)^ are not allowed in theory, though the algorithmic construction in Section 2.2 does not rule out their introduction. However, a rigorous understanding of the model in this setting remains challenging and constitutes an exciting perspective. In an effort to generate even more realistic image sequences, we may consider to blur the system of generated particles using the point spread function, and to add some noise and background, as carried out for instance in Ref. (8). In relation, additional features could be computed from both the real-image sequence and the synthetic ones in order to strengthen the quality assessment of the generator.

## References

[1] Kervrann C, Sorzano CÓS, Acton ST, Olivo-Marin J-C and Unser M (2015) A guided tour of selected image processing and analysis methods for fluorescence and electron microscopy. IEEE Journal of Selected Topics in Signal Processing 10(1), 6–30.

[2] Chenouard N, Smal I, De Chaumont F, Maška M, Sbalzarini IF, Gong Y, Cardinale J, Carthel C, Coraluppi S, Winter M, Cohen AR, Godinez WJ, Rohr K, Kalaidzidis Y, Liang L, Duncan J, Shen H, Xu Y, Magnusson KE, Jaldén J, Blau HM, Paul-Gilloteaux P, Roudot P, Kervrann C, Waharte F, Tinevez JY, Shorte SL, Willemse J, Celler K, van Wezel GP, Dan HW, Tsai YS, Ortiz de Solórzano C, Olivo-Marin JC and Meijering E (2014) Objective comparison of particle tracking methods. Nature Methods 11(3), 281–289. 24441936 10.1038/nmeth.2808 PMC4131736

[3] García-Fernández AF, Svensson L and Morelande MR (2020) Multiple target tracking based on sets of trajectories. IEEE Transactions on Aerospace and Electronic Systems 56(3), 1685–1707.

[4] Roudot P, Ding L, Jaqaman K, Kervrann C and Danuser G (2017) Piecewise-stationary motion modeling and iterative smoothing to track heterogeneous particle motions in dense environments. IEEE Transactions on Image Processing 26(11), 5395–5410. 29388914 10.1109/TIP.2017.2707803 PMC5796444

[5] Vo B-N, Vo B-T and Phung D (2014) Labeled random finite sets and the bayes multi-target tracking filter. IEEE Transactions on Signal Processing 62(24), 6554–6567.

[6] Badoual A, Arizono M, Denizot A, Ducros M, Berry H, Nägerl UV and Kervrann C (2021) Simulation of astrocytic calcium dynamics in lattice light sheet microscopy images. In 2021 IEEE 18th International Symposium on Biomedical Imaging (ISBI). Nice, France: IEEE, pp. 135–139.

[7] Bourgeois D (2023) Single molecule imaging simulations with advanced fluorophore photophysics. Communications Biology 6(1), 53. 36646743 10.1038/s42003-023-04432-x PMC9842740

[8] Lagardère M, Chamma I, Bouilhol E, Nikolski M and Thoumine O (2020) Fluosim: Simulator of single molecule dynamics for fluorescence live-cell and super-resolution imaging of membrane proteins. Scientific Reports 10(1), 1–14. 33203884 10.1038/s41598-020-75814-y PMC7672080

[9] Sage D, Pham T-A, Babcock H, Lukes T, Pengo T, Chao J, Velmurugan R, Herbert A, Agrawal A, Colabrese S, Wheeler A, Archetti A, Rieger B, Ober R, Hagen GM, Sibarita JB, Ries J, Henriques R, Unser M and Holden S (2019) Super-resolution fight club: Assessment of 2d and 3d single-molecule localization microscopy software. Nature Methods 16(5), 387–395. 30962624 10.1038/s41592-019-0364-4 PMC6684258

[10] Venkataramani V, Herrmannsdörfer F, Heilemann M and Kuner T (2016) Suresim: Simulating localization microscopy experiments from ground truth models. Nature Methods 13(4), 319–321. 26928761 10.1038/nmeth.3775

[11] Schaff J, Fink CC, Slepchenko B, Carson JH and Loew LM (1997) A general computational framework for modeling cellular structure and function. Biophysical Journal 73(3), 1135–1146. 9284281 10.1016/S0006-3495(97)78146-3 PMC1181013

[12] Gupta S, Czech J, Kuczewski R, Bartol TM, Sejnowski TJ, Lee RE and Faeder JR (2018) Spatial stochastic modeling with mcell and cellblender. *arXiv preprint arXiv:1810.00499.*

[13] Andrews SS, Addy NJ, Brent R and Arkin AP (2010) Detailed simulations of cell biology with smoldyn 2.1. PLoS Computational Biology 6(3), 1–10. 10.1371/journal.pcbi.1000705 PMC2837389 20300644

[14] Andrews SS (2018) Particle-based stochastic simulators. Encyclopedia of Computational Neuroscience 10, 978.

[15] Bolte S and Cordelieres F (2006) A guided tour into subcellular colocalization analysis in light microscopy. Journal of Microscopy 224, 213–232. 17210054 10.1111/j.1365-2818.2006.01706.x

[16] Costes S, Daelemans D, Cho E, Dobbin Z, Pavlakis G and Lockett S (2004) Automatic and quantitative measurement of protein-protein colocalization in live cells. Biophysical Journal 86(6), 3993–4003. 15189895 10.1529/biophysj.103.038422 PMC1304300

[17] Lagache T, Sauvonnet N, Danglot L and Olivo-Marin J-C (2015) Statistical analysis of molecule colocalization in bioimaging. Cytometry Part A 87(6), 568–579. 10.1002/cyto.a.22629 25605428

[18] Lavancier F, Pécot T, Zengzhen L and Kervrann C (2020) Testing independence between two random sets for the analysis of colocalization in bioimaging. Biometrics 76(1), 36–46. 31271216 10.1111/biom.13115

[19] Arts M, Smal I, Paul MW, Wyman C and Meijering E (2019) Particle mobility analysis using deep learning and the moment scaling spectrum. Scientific Reports 9(1), 17160. 31748591 10.1038/s41598-019-53663-8 PMC6868130

[20] Boulanger J, Kervrann C and Bouthemy P (2009) A simulation and estimation framework for intracellular dynamics and trafficking in video-microscopy and fluorescence imagery. Medical Image Analysis 13(1), 132–142. 18723385 10.1016/j.media.2008.06.017

[21] Bressloff PC and Newby JM (2013) Stochastic models of intracellular transport. Reviews of Modern Physics 85(1), 135.

[22] Briane V, Kervrann C and Vimond M (2018) Statistical analysis of particle trajectories in living cells. Physical Review E 97(6-1), 062121. 30011544 10.1103/PhysRevE.97.062121

[23] Briane V, Vimond M and Kervrann C (2019) An overview of diffusion models for intracellular dynamics analysis. Briefings in Bioinformatics 21, bbz052. 10.1093/bib/bbz052 31204428

[24] Hozé N and Holcman D (2017) Statistical methods for large ensembles of super-resolution stochastic single particle trajectories in cell biology. Annual Review of Statistics and Its Application 4, 189–223.

[25] Muñoz-Gil G, Volpe G, Garcia-March MA, Aghion E, Argun A, Hong CB, Bland T, Bo S, Conejero JA, Firbas N, Garibo i Orts Ò, Gentili A, Huang Z, Jeon J-H, Kabbech H, Kim Y, Kowalek P, Krapf D, Loch-Olszewska H, Lomholt MA, Masson J-B, Meyer PG, Park S, Requena B, Smal I, Song T, Szwabiński J, Thapa S, Verdier H, Volpe G, Widera A, Lewenstein M, Metzler R and Manzo C (2021) Objective comparison of methods to decode anomalous diffusion. Nature Communications 12(1), 6253. 10.1038/s41467-021-26320-w PMC8556353 34716305

[26] Pécot T, Zengzhen L, Boulanger J, Salamero J and Kervrann C (2018) A quantitative approach for analyzing the spatio-temporal distribution of 3d intracellular events in fluorescence microscopy. eLife 7, e32311. 30091700 10.7554/eLife.32311 PMC6085121

[27] Kervrann C (2022) Biomolecule trafficking and network tomography-based simulations. In Biomedical Image Synthesis and Simulation. Amsterdam: Elsevier, pp. 543–569.

[28] Lagache T, Dauty E and Holcman D (2009) Quantitative analysis of virus and plasmid trafficking in cells. Physical Review E 79(1), 011921. 10.1103/PhysRevE.79.011921 19257083

[29] Lavancier F and Le Guével R (2021) Spatial birth-death-move processes: Basic properties and estimation of their intensity functions. Journal of the Royal Statistical Society: Series B (Statistical Methodology) 83 4, 798–825.

[30] Boulanger J, Gueudry C, Munch D, Cinquin B, Paul-Gilloteaux P, Bardin S, Guérin C, Senger F, Blanchoin L and Salamero J (2014) Fast high-resolution 3D total internal reflection fluorescence microscopy by incidence angle scanning and azimuthal averaging. Proceedings of the National Academy of Sciences of the United States of America 111(48), 17164–17169. 25404337 10.1073/pnas.1414106111 PMC4260613

[31] Øksendal B (2013) Stochastic Differential Equations: An Introduction with Applications. Berlin: Springer Science & Business Media.

[32] Lavancier F, Le Guével R and Manent E (2022) Feller and ergodic properties of jump-move processes with applications to interacting particle systems. *arXiv preprint arXiv:2204.02851.*

[33] Pécot T, Bouthemy P, Boulanger J, Chessel A, Bardin S, Salamero J and Kervrann C (2014) Background fluorescence estimation and vesicle segmentation in live cell imaging with conditional random fields. IEEE Transactions on Image Processing 24(2), 667–680. 25531952 10.1109/TIP.2014.2380178

[34] Pécot T, Kervrann C, Bardin S, Goud B and Salamero J (2008) Patch-based markov models for event detection in fluorescence bioimaging. In International Conference on Medical Image Computing and Computer-Assisted Intervention. Berlin: Springer, pp. 95–103. 10.1007/978-3-540-85990-1_12 18982594

[35] Jaqaman K, Loerke D, Mettlen M, Kuwata H, Grinstein S, Schmid SL and Danuser G (2008) Robust single-particle tracking in live-cell time-lapse sequences. Nature Methods 5(8), 695. 18641657 10.1038/nmeth.1237 PMC2747604

[36] Liptser RS and Shiryaev AN (2013) *Statistics of Random Processes II: Applications*, vol. 6. Cham: Springer Science & Business Media.

[37] Löcherbach E (2002) LAN and LAMN for systems of interacting diffusions with branching and immigration. In Annales de l’Institut Henri Poincare (B) Probability and Statistics, vol. 38. Amsterdam: Elsevier, pp. 59–90.

[38] Löcherbach E (2002) Likelihood ratio processes for markovian particle systems with killing and jumps. Statistical Inference for Stochastic Processes 5(2), 153–177.

[39] Korabel N, Waigh TA, Fedotov S and Allan VJ (2018) Non-markovian intracellular transport with sub-diffusion and run-length dependent detachment rate. PLoS One 13(11), 1–23. 10.1371/journal.pone.0207436 PMC6261056 30475848

